# Disrupted Topological Organization of Brain Network in Rats with Spatial Memory Impairments Induced by Acute Microwave Radiation

**DOI:** 10.3390/brainsci13071006

**Published:** 2023-06-28

**Authors:** Haoyu Wang, Haixia Zhao, Chunfang Li, Ji Dong, Jianghao Zhao, Hanlin Yue, Yunfei Lai, Li Zhao, Hui Wang, Jing Zhang, Xinping Xu, Binwei Yao, Hongmei Zhou, Binbin Nie, Xiumin Du, Ruiyun Peng

**Affiliations:** 1 Beijing Institute of Radiation Medicine, Beijing 100850, China; smart106@126.com (H.W.);; 2 College of Education, Hebei University, Baoding 071002, China; 3 Department of Radiology, The First Medical Center, Chinese PLA General Hospital, Beijing 100853, China; 4 Beijing Engineering Research Center of Radiographic Techniques and Equipment, Institute of High Energy Physics, Chinese Academy of Sciences, Beijing 100049, China; niebb@ihep.ac.cn

**Keywords:** microwave radiation, topological organization, brain network, spatial memory, resting-state fMRI, rat

## Abstract

Previous studies have suggested that microwave (MW) radiation with certain parameters can induce spatial memory deficits. However, the effect of MW on the topological organization of the brain network is still unknown. This work aimed to investigate the topological organization of the brain network in rats with spatial memory impairments induced by acute microwave (MW) radiation. The Morris water maze (MWM) test and resting-state functional magnetic resonance imaging were performed to estimate the spatial memory ability and brain network topological organization of the rats after MW exposure. Compared with the sham group, the rats exposed to 30 mW/cm^2^ 1.5 GHz MW radiation exhibited a significantly decreased normalized clustering coefficient (γ) (*p* = 0.002) 1 d after the exposure and a prolonged average escape latency (AEL) (*p* = 0.014) 3 d after the exposure. Moreover, after 10 mW/cm^2^ 1.5 GHz MW radiation, a significantly decreased γ (*p* = 0.003) was also observed in the rats, without any changes in AEL. In contrast, no adverse effects on AEL or topological parameters were observed after 9.375 GHz MW radiation. In conclusion, the rats with spatial memory deficits induced by MW radiation exhibited disruptions in the topological organization of the brain network. Moreover, these topological organization disruptions emerged earlier than behavioral symptom onset and could even be found in the rats without a decline in the performance of the spatial memory task. Therefore, it is possible to use the topological parameters of the brain network as early and sensitive indicators of the spatial memory impairments induced by acute MW radiation.

## 1. Introduction

Microwave (MW) refers to the electromagnetic field with frequencies ranging from 300 MHz to 300 GHz. MW has been widely used in industry, medicine, the military, and household living [1,2]. Consequently, a large portion of the global population is now subjected to MW either in their daily lives or in particular occupational environments. Concurrent with its broad applications, the effects of MW on human health have also roused attention [3,4]. The L band (1 GHz–2 GHz) and X band (8 GHz–12 GHz) are two of the most commonly used MW bands in telecommunications. Previous studies have demonstrated that both L-band and X-band MW radiation with certain parameters can cause detrimental effects on cognitive functions, especially spatial memory ability [5,6,7,8]. For example, Wang et al. [8] found that acute exposure (5 min) to 1.5 GHz microwave with an average power density of 30 mW/cm^2^ could induce spatial memory dysfunction in rats.

Resting-state functional magnetic resonance imaging (rs-fMRI) is one of the most popular non-invasive techniques widely used in investigations of cognitive deficits induced by various diseases such as Alzheimer’s disease (AD) [9,10], Parkinson’s disease [11], and schizophrenia [12]. rs-fMRI has also been used in previous studies to investigate the effects of MW on brain functions [13,14]. These pioneering works mainly focused on regional brain activities and inter-regional functional connectivity, defined as temporal dependency between neurophysiological activities in spatially remote brain regions. Using rs-fMRI, Lv and colleagues [15] found that the amplitudes of low-frequency fluctuation and fractional amplitudes of low-frequency fluctuation in specific human brain regions significantly decreased after MW exposure. By implementing the regional homogeneity method and seed-based analysis based on rs-fMRI data, Wei et al. [16] also demonstrated that acute MW exposure can alter both localized intra-regional and inter-regional functional connectivity. However, the brain actually works as a tightly integrated and highly efficient network, rather than operating through the activities of isolated brain regions or inter-regional functional connections between specific brain regions. Examinations of the properties of this functional brain network can provide us with new insights into large-scale neuronal communication changes in the brain after MW exposure [17].

Based on graph theory, it has been suggested that the functional brain network has the topological organization of a small-world network, which can maintain an optimal balance between the separation and integration of information with low energy and wiring costs and is suited for complex brain dynamics [18,19]. Using neuroimaging techniques, several resting-state brain networks, including the motor network [20,21,22], visual network [23], default mode network (DMN) [24,25,26,27,28], etc., have been found in human brains. In rodents, resting-state brain networks have also been reported [29,30]. A DMN-like resting-state brain network consisting of the cingulate, retrosplenial, parietal, orbital, prelimbic, and auditory/temporal-associated cortices and the hippocampus was found in rats [31,32]. Moreover, Wang et al. [33] proposed a resting-state sensory–cognitive network in rats consisting of the retrosplenial cortex, parietal association cortex, hippocampus, and thalamus nucleus. They suggested that this resting-state brain network was responsible for processing sensory information, spatial learning, and episodic memory. Over the past few decades, mounting evidence has demonstrated that there exist strong links between the topological organization disruptions of the resting-state brain network and memory impairments induced by various diseases such as AD [34], schizophrenia [35], chronic post-traumatic stress disorder [36,37], end-stage renal disease [38], hyperthyroidism [39], and type 2 diabetes mellitus [40]. However, whether there exists a disruption of the topological organization of the whole-brain functional network in rats with acute-MW-radiation-induced spatial memory impairments remains unclear.

In this study, we hypothesized that changes in the topological organization of the brain network are present in rats with spatial memory impairments induced by acute MW radiation. To test our hypotheses, the Morris water maze (MWM) test was used to measure the spatial memory ability of rats after MW exposure. Moreover, graph theory was applied to investigate the topological organization of the brain network in the rats after MW exposure based on rs-fMRI data.

## 2. Materials and Methods

### 2.1. Animals and Groups

A total of 85 male specific-pathogen-free (SPF) Wistar rats (8 weeks, 220~230 g) (Beijing Vital River Laboratory Animal Technology Co., Ltd., Beijing, China) were housed in a constant environment (24 ± 2 °C, 12-h light/dark cycle, 50% relative humidity) with ad libitum access to food and water. The rats were randomly divided into 5 groups (*n* = 17 per group, 12 for MWM, 5 for rs-fMRI): (1) a sham group; (2) a 10 mW/cm^2^ 1.5 GHz MW exposure group (L10 group); (3) a 30 mW/cm^2^ 1.5 GHz MW exposure group (L30 group); (4) a 10 mW/cm^2^ 9.375 GHz MW exposure group (X10 group); and (5) a 30 mW/cm^2^ 9.375 GHz MW exposure group (X30 group).

### 2.2. MW Exposure

Two MW simulators (developed by the Beijing Institute of Radiation Medicine) with center frequencies of 1.5 GHz and 9.375 GHz were used in this study. The exposure systems transmitted MW energy through rectangular waveguides and horn antennas, as described in previous studies [41,42].

**Figure 1:**
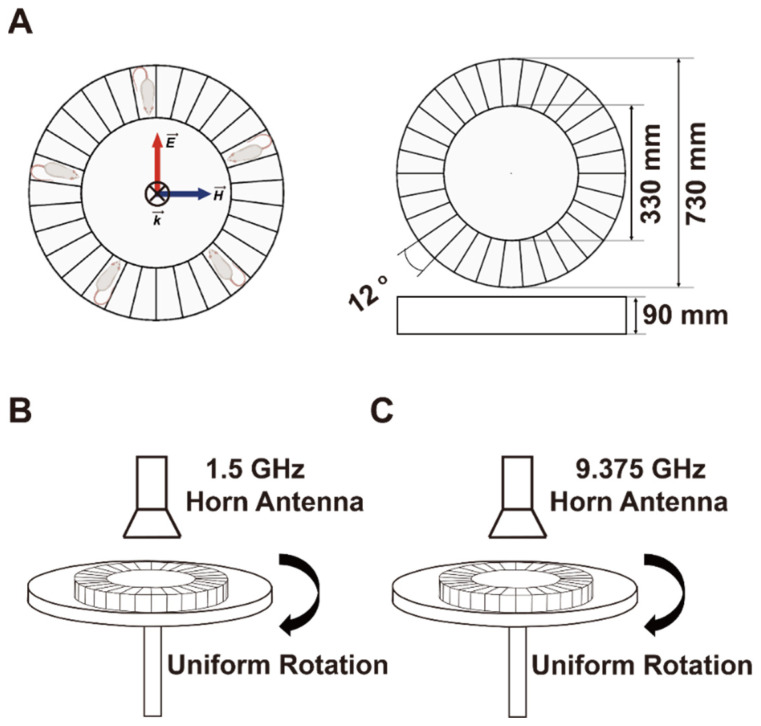
Schematic diagram of the MW. (**A**) shows a schematic diagram of the tailor-made rat container and the orientations of the rats within it. The directions of the wave vector $\vec{k}$ is indicated by the cross; the electric field $\vec{E}$ is indicated by the red arrow; the magnetic field $\vec{H}$ is indicated by the blue arrow. (**B**,**C**) show schematic diagrams of the 1.5 GHz and 9.375 GHz MW processes, respectively. Abbreviations: MW, microwave.

The rats were placed in a metal-free fixation container made of plexiglass (Figure 1A). Then, the container was placed on the radiation platform of the MW simulator system (Figure 1B,C). For the exposure groups, the rats were whole-body exposed to MW for 10 min. The incident direction of the MW was from the dorsal side to the ventral side of the rats [43]. During exposure, the radiation platform rotated at a constant speed (180° per min) to ensure that each rat received the same dose (Figure 1B,C). For the sham group, the rats were treated in the same way as the other groups, except for the lack of MW exposure.

### 2.3. Dosimetric Analysis

As shown in Figure 1A, in our realistic exposure experiments, the incident direction (i.e., the direction of the wave vector $\vec{k}$, indicated by a cross in Figure 1A) of the MW was perpendicular to the exposure platform. Since the rats were constrained in a prone position in the fixation container, the incident direction of the MW (either 1.5 GHz or 9.375 GHz) was from the dorsal side to the ventral side of the rats. Meanwhile, both the E-polarization direction (i.e., the direction of electric field $\vec{E}$, indicated by a red arrow in Figure 1A) of the MW and the longitudinal axis of the rats were in the plane of the platform surface. Thus, the longitudinal axis of the rat formed an angle *θ* with the E-polarization direction of the MW (hereafter referred to as “E-polarization angle *θ*”) when the rat rotated along with the radiation platform to a certain position.

**Figure 2:**
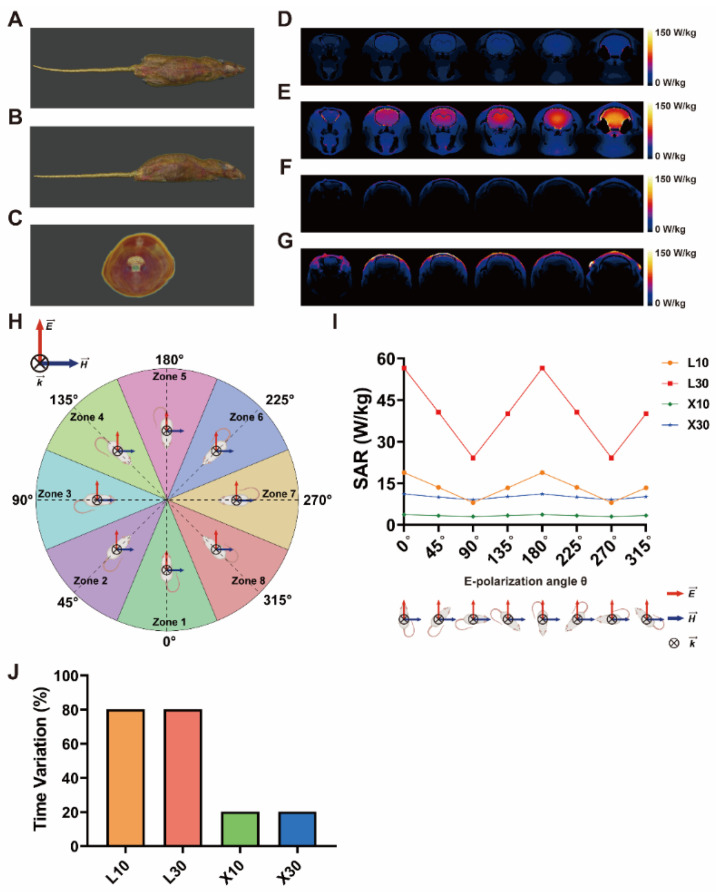
Spatial distributions of and temporal variations in SAR values of rat brains. (**A**–**C**) show the top view, lateral view, and front view of the numerical voxel model of rats with 47 different tissues, respectively. (**D**–**G**) demonstrate the spatial distributions of the SAR values of rat brains in the 10 mW/cm^2^ 1.5 GHz MW exposure group (L10 group), 30 mW/cm^2^ 1.5 GHz MW exposure group (L30 group), 10 mW/cm^2^ 9.375 GHz MW exposure group (X10 group), and 30 mW/cm^2^ 9.375 GHz MW exposure group (X30 group), respectively. The color bars denote the SAR values. (**H**) shows a schematic diagram of the circular exposure area division. The red arrow, blue arrow, and cross denote the electric field $\vec{E}$, the magnetic field $\vec{H}$, and the incident wave vector $\vec{k}$, which was pointing into the page, respectively. The circular exposure area was equally divided into eight zones (Zone 1–Zone 8) indicated by different colors. Eight representative center E-polarization angles (0°, 45°, 90°, 135°, 180°, 225°, 270°, and 315°) were selected in each zone to estimate the average SAR values of rat brains in the special zone during the exposure. (**I**,**J**) show the temporal variations in the SAR values of rat brains during MW exposure caused by the rotation of the platform. Abbreviations: SAR, specific absorption rate; MW, microwave.

In this work, the energy deposit in the rat brain was evaluated using the SAR value based on a numerical rat model with 47 different tissues (Figure 2A–C). The SAR value of a specific tissue *t* at the E-polarization angle *θ* can be calculated using the following formula:

(1) $${\mathrm{SAR}}_{t,\theta }=\frac{\sigma_{t}\cdot {\left|\vec{E_{t,\theta }}\right|}^{2}}{\rho_{t}},$$

where *σ_t_* denotes the electric conductivity (S/m) of the specific tissue *t*; ρ*_t_* denotes the density (kg/m^3^) of the tissue *t*; $\left|\vec{E_{t,\theta }}\right|$ denotes the electric field strength in the tissue *t* at the E-polarization angle *θ*.

Considering the scenarios of our exposure experiments, the realistic average SAR value of rat brains $\bar{{SAR}_{brain}}$ during the MW exposure was calculated as described below. The circular exposure area could be equally divided into *N* fan-shaped zones. Figure 2H shows a schematic diagram of the circular exposure area division when *N* = 8. The realistic average SAR value of the rat brains $\bar{{SAR}_{brain}}$ during the MW exposure can be calculated as a weighted average of the SAR values of each zone:

(2) $$\bar{{SAR}_{brain}}=\sum_{i}^{N}(\bar{{SAR}_{brain,i}}\times {TW}_{i}),$$

where *N* denotes the number of fan-shaped zones uniformly divided in the circular exposure area; $\bar{{SAR}_{brain,i}}$ denotes the average SAR value of the rat brains within the *i*th zone (for *i* = 1, 2, …, *N*); ${TW}_{i}$ denotes the time weight of the *i*th zone (for *i* = 1, 2, …, *N*), which is defined as follows:

(3) $${TW}_{i}=\frac{{Time}_{i}}{{Time}_{total}},$$

where ${Time}_{i}$ denotes the time that rats spent in the *i*th zone (for *i* = 1, 2, …, *N*); ${Time}_{total}$ is the total exposure duration time calculated as the sum of ${Time}_{i}$:

(4) $${Time}_{total}=\sum_{i}^{N}{Time}_{i}.$$

Since the rotation speed of the radiation platform was constant (180° per min), the time that the rats spent in each zone was the same. Therefore, ${TW}_{i}$ = 1/*N* (for *i* = 1, 2, …, *N*). Then, Equation (2) can be rewritten as follows:

(5) $$\bar{{SAR}_{brain}}=\frac{1}{N}\sum_{i}^{N}\bar{{SAR}_{brain,i}}.$$

The average SAR value of the rat brains within the *i*th zone $\bar{{SAR}_{brain,i}}$ (for *i* = 1, 2, …, *N*) can be calculated as the weighted average of the SAR values of the rat brains at each E-polarization angle,

(6) $$\bar{{SAR}_{brain,i}}=\sum_{k}^{M}({SAR}_{brain,\theta_{i,k}}\times {TW}_{\theta_{i,k}}),$$

where *M* denotes the number of angles within the *i*th zone; ${SAR}_{brain,\theta_{i,k}}$ denotes the SAR value of the rat brain estimated at the *k*th E-polarization angle *θ_i_*_,*k*_ (for *k* = 1, 2, …, *M*) in the *i*th zone; ${TW}_{\theta_{i,k}}$ denotes the time weight of the *k*th E-polarization angle *θ_i,k_* (for *k* = 1, 2, …, *M*) in the *i*th zone, which is defined as follows:

(7) $${TW}_{\theta_{i,k}}={Time}_{\theta_{i,k}}/{Time}_{i},$$

where ${Time}_{\theta_{i,k}}$ denotes the time that rats spent at the *k*th E-polarization angle *θ_i,k_* (for *k* = 1, 2, …, *M*) in the *i*th zone; ${Time}_{i}$ denotes the time that rats spent in the *i*th zone (for *i* = 1, 2, …, *N*), which equals the sum of ${Time}_{\theta_{i,k}}$:

(8) $${Time}_{i}=\sum_{k}^{M}{Time}_{\theta_{i,k}}.$$

Since the rotation speed of the platform was constant, the time that the rats spent at each E-polarization angle was the same. Therefore, ${TW}_{\theta_{i,k}}$ = 1/*M* (for *k* = 1, 2, …, *M*). Then, Equation (6) can be rewritten as follows:

(9) $$\bar{{SAR}_{brain,i}}=\frac{1}{M}\sum_{k}^{M}{SAR}_{brain,\theta_{i,k}},$$

When the zone is small, the SAR values of rat brains at different E-polarization angles within the zone are assumed to be the same as that obtained at the center E-polarization angle of the zone. For the *i*th zone,

(10) $${SAR}_{brain,\theta_{i,k}}={SAR}_{brain,\theta_{i,center}},$$

where ${SAR}_{brain,\theta_{i,center}}$ denotes the SAR value of the rat brain at the center E-polarization angle $\theta_{i,center}$ of the *i*th zone.

Substituting Equation (10) into Equation (9),

(11) $$\bar{{SAR}_{brain,i}}={SAR}_{brain,\theta_{i,center}},$$

Substituting Equation (11) into Equation (5),

(12) $$\bar{{SAR}_{brain}}=\frac{1}{N}\sum_{i}^{N}{SAR}_{brain,\theta_{i,center}}.$$

The SAR value of the rat brain at the center E-polarization angle $\theta_{i,center}$ of the *i*th zone ${SAR}_{brain,\theta_{i,center}}$ can be calculated using Equation (1).

In this study, to approximately estimate the average SAR value of the rat brains, our simulation experimental protocol was as follows. Firstly, a numerical rat model consisting of 47 different tissues was imported in the simulation. Secondly, a plane wave MW source with a certain combination of physical parameters (one of these combinations: 10 mW/cm^2^–1.5 GHz MW, 30 mW/cm^2^–1.5 GHz MW, 10 mW/cm^2^–9.375 GHz MW, or 30 mW/cm^2^–9.375 GHz MW) was used to mimic the MW radiation. The incident direction of the MW was set to be from the dorsal side to the ventral side of the rats (Figure 2H). Thirdly, we equally divided the circular exposure area into 8 fan-shaped zones. The center E-polarization angles of these zones were 0°, 45°, 90°, 135°, 180°, 225°, 270°, and 315°, respectively (Figure 2H). According to Equation (12), the approximate averaged SAR value $\bar{{SAR}_{brain}}\prime$ in the rat brain during the exposure can be calculated as follows:

(13) $$\bar{{SAR}_{brain}}\prime =\frac{1}{8}\sum_{i}^{8}{SAR}_{brain,\theta_{i,center}},$$

where $\theta_{1,center}$ = 0°, $\theta_{2,center}$ = 45°, $\theta_{3,center}$ = 90°, $\theta_{4,center}$ = 135°, $\theta_{5,center}$ = 180°, $\theta_{6,center}$ = 225°, $\theta_{7,center}$ = 270°, and $\theta_{8,center}$ = 315°. In the simulation environment, eight separate simulations were performed. For each simulation, the E-polarization angle was adjusted to one of the center E-polarization angles. The ${SAR}_{brain,\theta_{i,center}}$ was estimated based on Equation (1) using the finite difference time domain (FDTD) method. The dielectric properties of different tissues at either 1.5 GHz or 9.375 GHz were in accordance with the IT’IS database Version 4.1. Finally, the approximate average SAR value of a rat brain during the MW exposure $\bar{{SAR}_{brain}}\prime$ can be obtained using Equation (13).

Moreover, the temporal variations in the SAR values of the rat brains between different E-polarization directions during rotation were calculated using the following formula:

(14) $$Variation=\frac{{SAR}_{max}-{SAR}_{min}}{{SAR}_{mean}}\times 100\%,$$

where SAR*_max_*, SAR*_min_*, and SAR*_mean_* denoted the maximum, minimum, and averaged SAR values across the 8 selected E-polarization angles.

All simulations were conducted using a workstation with the following configurations: CPU: Core™ i7-11700K: 3.6 GHz (Intel, Santa Clara, CA, USA), RAM: 16 GB, GPU: UHD Graphics 750 (Intel, Santa Clara, CA, USA).

### 2.4. Morris Water Maze (MWM)

The MWM is a behavioral test designed for the evaluation of rodent spatial memory [44]. Rats participating in MWM need to find the location of the submerged escape platform using distal cues around the open swimming arena. In this study, a black circular pool with a diameter of 1.5 m was used as the open swimming arena. The pool was divided into 4 equal quadrants filled with 20 ± 1 °C clear water and surrounded by curtains. The escape platform was also black, which created a nearly invisible platform-to-background color match. The diameter of the escape platform was 12 cm, and it was placed in one of the quadrants and submerged 1 cm below the water surface.

Before MW exposure, all 60 rats (*n* = 12 for each group) were trained in 4 trials per day for 3 days. The rats were placed in the pool in 4 different quadrants in a semi-random sequence, as described in the previous literature [44]. For each rat, the interval required between 2 successive trials to restore the rat’s physical strength was more than 30 min. During the training stage, if the rat found the platform within 60 s, it was allowed to stay on the platform for 15 s. If not, the rat was guided to the platform to stay there for 15 s.

On days 1–3 and day 7 after exposure, the navigation test was performed to evaluate the spatial memory abilities of the rats. In the navigation test, the average escape latency (AEL), defined as the average time that the rat took to reach the escape platform in all the trials (if the rat did not find the platform within 60 s, the escape latency was recorded as 60 s), was measured. Moreover, the swimming speed of each rat was also calculated to evaluate its locomotion ability. In the MWM test, ANYmaze 6.0 (Stoelting, Kiel, WI, USA) was used to monitor the whole procedure for each rat.

### 2.5. rs-fMRI Data Acquisition and Analysis

The rs-fMRI data of the rats (*n* = 5 for each group) were obtained using a 3.0-T MRI scanner (Discovery 750 MR, GE Healthcare, Chicago, IL, USA). During MRI scanning, the rats were anesthetized with an intraperitoneal injection of 1% pentobarbital sodium. Multi-slice coronal T2-weighted images were obtained using a periodically rotated overlapping parallel lines with enhanced reconstruction (PROPELLER) protocol with the following parameters: field of view = 100 mm × 100 mm, matrix size = 512 × 512, repetition time (TR) = 8083 ms, echo time (TE) = 160 ms, slice thickness = 1.5 mm, without a slice gap. The rs-fMRI data were obtained using an echo-planar imaging (EPI) pulse sequence with the following parameters: field of view = 50 mm × 50 mm, matrix size = 64 × 64, repetition time (TR) = 2009 ms, echo time (TE) = 31.2 ms, slice thickness = 1.5 mm, without a slice gap, number of time points = 200.

**Figure 3:**
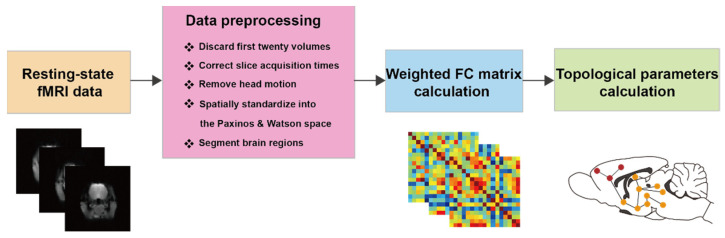
Flowchart for brain network analysis process.

After data acquisition, the rs-fMRI data were preprocessed using Data Processing Assistant for Resting-State fMRI (DPARSF) [45] with the following main steps. Firstly, the first twenty volumes of each rat were discarded to allow for magnetization equilibrium. Then, the differences in the slice acquisition times for each individual were corrected using slice timing, and the temporal processed volumes of each subject were realigned to the first volume to remove the head motion. All rats had less than 1 mm of translation in the *x*, *y*, or *z* axis and 1° of rotation in each axis. The realigned volumes were spatially standardized into the Paxinos and Watson space according to the rat brain division template provided by the spmratIHEP software [46]. Then, the brain regions including the retrosplenial cortex (RSC), parietal cortex (PC), hippocampus (Hip), dentate gyrus (DG), thalamus (Th), mammillary nuclei (Mm), primary somatosensory cortex (S1), secondary somatosensory cortex (S2), cingulate cortex (Cg), entorhinal cortex (Ent), piriform cortex (Pir), insular cortex (IC), motor cortex (M1), visual cortex (V1), amygdala (Am), habenula (Hb), and lateral septal nucleus (LS) of the rats were segmented using the spmratIHEP software. The weighted FC matrix was calculated based on an analysis of the Pearson correlations between changes in the resting-state BOLD-fMRI signals among these brain regions. The resting-state functional brain network was constructed on the macroscale, in which the nodes represented the brain regions and edges represented the interregional FC. Finally, based on the weighted FC matrices, the topological parameters of the functional brain network were calculated over sparsity values ranging from 0.15 to 0.5 to ensure high correlation coefficients of the remaining connections. The topological parameters (global efficiency, E_g_; local efficiency, E_loc_; clustering coefficient, C_p_; characteristic path length, L_p_; normalized clustering coefficient, γ; normalized characteristic path length, λ; and small-worldness, σ) of all the rats were calculated using Graph Theoretical Network Analysis (Gretna, Imaging Connectomics Lab at Beijing Normal University, Beijing, China) based on a small-world model with the FC matrix as the input (Figure 3). E_g_, E_loc_, C_p_, and L_p_ were defined as described in the previous studies [47,48,49]. Essentially, E_g_ measures the global efficiency of parallel information transfer in the network, and E_loc_ reveals the network fault-tolerance level. C_p_ indicates the extent of local interconnectivity in a network, and L_p_ quantifies the extent of the overall communication efficiency of a network. Moreover, γ is defined as the ratio between C_p_ and C_p-rand_ (γ = C_p_/C_p-rand_), where C_p-rand_ represents the mean C_p_ of the matched random networks (having low C_p_). λ is defined as the ratio between L_p_ and L_p-rand_ (λ = L_p_/L_p-rand_), where L_p-rand_ represents the mean L_p_ of the matched random networks (having high L_p_). Furthermore, the small-worldness σ is defined as the ratio between γ and λ, which can collectively reflect γ and λ. These three parameters (γ, λ, and σ) indicate the degree of small-world organization, which reflects an optimal balance of integration and segregation in a network [50,51,52,53]. For each topological parameter, the area under the curve (AUC) was used to obtain summary measures for the network properties across the full range of sparsity thresholds (0.15~0.5), with a step size of 0.05.

### 2.6. Statistical Analysis

All results are presented as the mean ± SEM in the paper. To investigate the effects of 1.5 GHz MW with different doses (10 mW/cm^2^ and 30 mW/cm^2^) on the spatial memory abilities of rats, a one-way ANOVA with post hoc tests was performed among the sham, L10, and L30 groups. Meanwhile, to investigate the effects of 1.5 GHz MW with different doses on the topological organization of the rat brain network, a one-way ANOVA with post hoc tests was performed among the sham, L10, and L30 groups. The *p*-values of the topological parameters were adjusted for multiple testing using Benjamini–Hochberg False Discovery Rate (FDR). Moreover, to investigate the effects of 9.375 GHz MW with different doses on the spatial memory abilities of rats, a one-way ANOVA with post hoc tests was performed among the sham, X10, and X30 groups. To investigate the effects of 9.375 GHz MW with different doses on the topological organization of the rat brain network, a one-way ANOVA with post hoc tests was performed among the sham, X10, and X30 groups. The *p*-values of the topological parameters were adjusted for multiple testing using Benjamini–Hochberg FDR.

For all measurements, the statistical significance was set to *p <* 0.05 (95% confidence interval), with * indicating *p <* 0.050 and ** indicating *p <* 0.010. All analyses were performed using IBM SPSS Statistics Version 19 (IBM, New York, NY, USA).

## 3. Results

### 3.1. Dosimetric Analysis

To investigate the energy deposits in the rat brains, a dosimetric analysis based on a digital rat model was performed. Table 1 lists the SAR values of rat brains at different center E-polarization angles and the approximate average brain SAR values $\bar{{SAR}_{brain}}\prime$ of different exposure groups. These results demonstrate that the approximate averaged SAR value of the rat brains was positively related to the average power density of MW and negatively related to the frequency of MW.

Figure 2 shows the spatial distributions of, and temporal variations in, the SAR values of rat brains under MW exposure with different frequencies and different E-polarization directions. As shown in Figure 2D–G, the spatial distributions of the SAR values varied at different frequencies. Specifically, for 1.5 GHz and 9.375 GHz MW, the energy was mainly deposited in the brain and skin, respectively. Figure 2I,J shows the temporal variations in the SAR values during one full rotation. It is clear that the temporal variations in the SAR values of 1.5 GHz MW exposure were much higher than those of 9.375 GHz MW exposure. These results suggest that both the spatial distribution of and temporal variation in the SAR values of the rat brains depended on the frequency of MW. Moreover, 1.5 GHz MW radiation led to higher doses of and larger temporal variations in energy deposits in the rat brains compared with 9.375 GHz MW.

### 3.2. Acute Exposure to 30 mW/cm^2^ 1.5 GHz MW Induced Spatial Memory Decline in Rats

**Figure 4:**
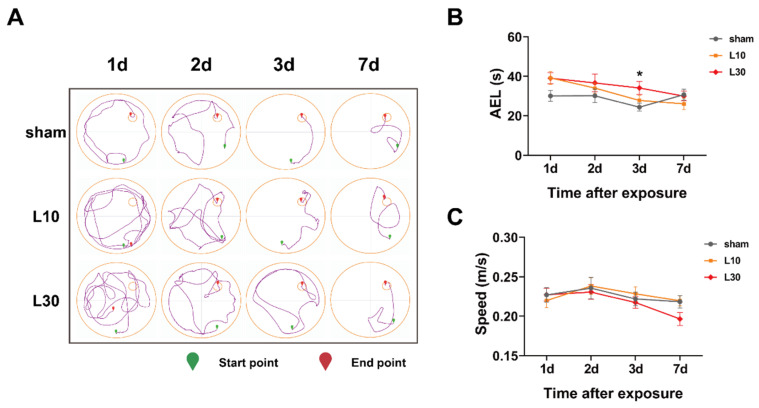
The effects of acute 1.5 GHz MW exposure on the spatial memory of rats. (**A**) shows the representative motion trajectories of rats in different groups in the MWM test (sham, L10, and L30) 1 d, 2 d, 3 d, and 7 d after MW exposure. (**B**,**C**) show comparisons of the AEL and swimming speed of rats in different groups in the MWM test (sham, L10, and L30) 1 d, 2 d, 3 d, and 7 d after MW exposure, respectively. Data are shown as mean ± SEM. * *p* < 0.05, L30 vs. sham. Abbreviations: MW, microwave; MWM, Morris water maze; AEL, average escape latency.

To investigate the effects of acute 1.5 GHz MW exposure on the spatial memory of rats, the MWM test was performed. The impacts of 1.5 GHz MW exposure on the average escape latency (AEL) of rats were evaluated. On the first two days after 10 mW/cm^2^ and 30 mW/cm^2^ 1.5 GHz MW exposure, no significant differences in the AEL among the sham, L10, and L30 groups were observed. On the thirdday after the MW exposure, significant AEL differences were observed in the L10 and L30 groups vs. that in the sham group [F (2, 33) = 3.465, *p* = 0.043]. As shown in Figure 4B, the post hoc test demonstrated that the AEL of rats in the L30 group was significantly prolonged (34.133 ± 3.281 s, *p* = 0.014) compared with that in the sham group (24.329 ± 2.000 s). Seven days after the MW exposure, no significant difference in the AEL among the sham, L10, and L30 groups was observed.

In order to exclude the possibility that the prolonged AEL was due to a difference in locomotor ability, the swimming speed of rats during the MWM test was also compared among different groups. As shown in Figure 4C, on 1 d, 2 d, 3 d, or 7 d after 10 mW/cm^2^ and 30 mW/cm^2^ 1.5 GHz MW exposure, there were no significant differences in swimming speed among the sham, L10, and L30 groups.

Taken together, these results suggest that acute exposure to 30 mW/cm^2^ 1.5 GHz MW could induce rat spatial memory impairment 3 d after the exposure. This adverse effect was recovered by 7 d after the exposure. Moreover, acute exposure to 10 mW/cm^2^ 1.5 GHz MW induced no adverse effect on the spatial memory of rats.

### 3.3. Acute Exposure to 10 mW/cm^2^ or 30 mW/cm^2^ 9.375 GHz MW Induced No Adverse Effect on Spatial Memory in Rats

**Figure 5:**
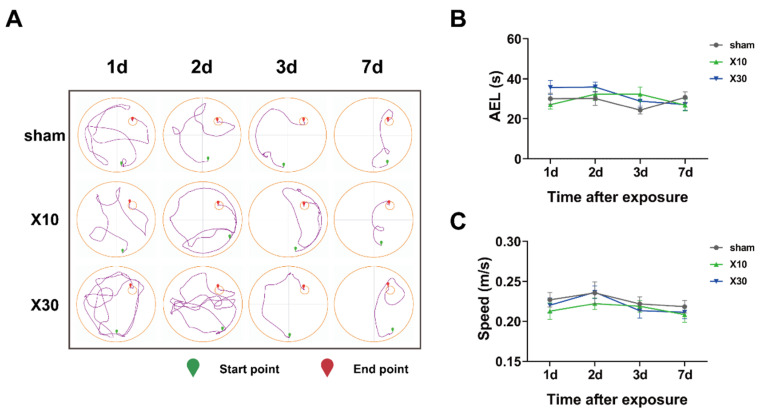
The effects of acute 9.375 GHz MW exposure on the spatial memory of rats. (**A**) shows the representative motion trajectories of rats in different groups in the MWM test (sham, X10, and X30) 1 d, 2 d, 3 d, and 7 d after MW exposure. (**B**,**C**) show comparisons of the AEL and swimming speed of rats in different groups in the MWM test (sham, X10, and X30) 1 d, 2 d, 3 d, and 7 d after MW exposure, respectively. Data are shown as mean ± SEM. Abbreviations: MW, microwave; MWM, Morris water maze; AEL, average escape latency.

To investigate the effects of acute 9.375 GHz MW exposure on the spatial memory of rats, the MWM test was also performed. As illustrated in Figure 5A,B, on 1 d, 2 d, 3 d, or 7 d after 10 mW/cm^2^ and 30 mW/cm^2^ 9.375 GHz MW exposure, there were no significant differences in the AEL among the sham, X10, and X30 groups. Meanwhile, the swimming speed values during the MWM test were comparable in rats from different groups. As shown in Figure 5C, on the first, second, third, and seventh day after the MW exposure, no significant AEL differences were observed in the L10 and L30 groups vs. that in sham group. These results suggest that acute exposure to either 10 mW/cm^2^ or 30 mW/cm^2^ 9.375 GHz MW induced no adverse effect on the spatial memory of rats.

### 3.4. Acute Exposure to 10 mW/cm^2^ and 30 mW/cm^2^ 1.5 GHz MW Induced Disrupted Topological Organization of the Brain Network in Rats

**Figure 6:**
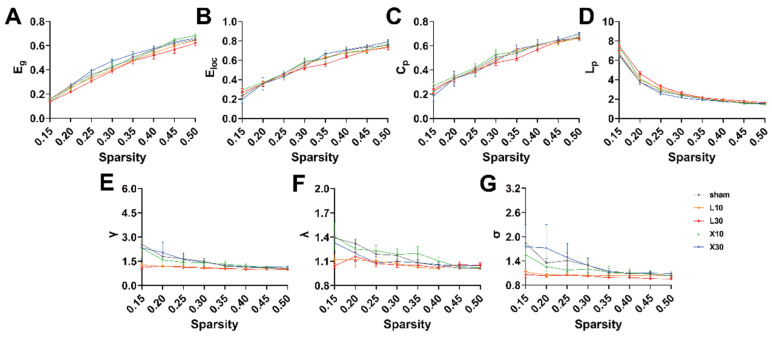
The changes in the brain network topological properties with the change in the sparsity threshold 1 d after acute 1.5 GHzMW exposure. (**A**–**G**) show the comparisons of the topological parameters (E_g_, E_loc_, C_p_, L_p_, γ, λ, and σ) of the rat brain network 1 d after MW exposure across the whole range of sparsity values (from 0.15 to 0.5 with an interval of 0.05) for different groups (sham, L10, L30, X10, and X30 groups, *n* = 5). Abbreviations: MW, microwave; E_g_, global efficiency; E_loc_, local efficiency; C_p_, clustering coefficient; L_p_, characteristic path length; γ, normalized clustering coefficient; λ, normalized characteristic path length; σ, small-worldness.

**Figure 7:**
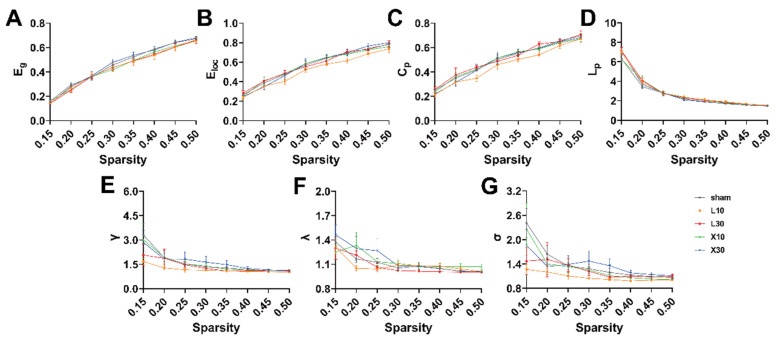
The changes in the brain network topological properties with the change in the sparsity threshold 7 d after acute 1.5 GHz MW exposure. (**A**–**G**) show the comparisons of the topological parameters (E_g_, E_loc_, C_p_, L_p_, γ, λ, and σ) of the rat brain network 7 d after MW exposure across the whole range of sparsity values (from 0.15 to 0.5 with an interval of 0.05) for different groups (sham, L10, L30, X10, and X30 groups, *n* = 5). Abbreviations: MW, microwave; E_g_, global efficiency; E_loc_, local efficiency; C_p_, clustering coefficient; L_p_, characteristic path length; γ, normalized clustering coefficient; λ, normalized characteristic path length; σ, small-worldness.

rs-fMRI was used to investigate the changes in the topological organization of the rat brain network 1 d and 7 d after MW exposure. One day after 10 mW/cm^2^ and 30 mW/cm^2^ 1.5 GHz MW exposure, across the whole range of sparsity thresholds, lower γ values were observed in the L10 and L30 groups compared with those in the sham group (Figure 6E,G). Seven days after the exposure, the decreases in the γ values of rats in the L10 and L30 groups were alleviated (Figure 7E,G).

**Figure 8:**
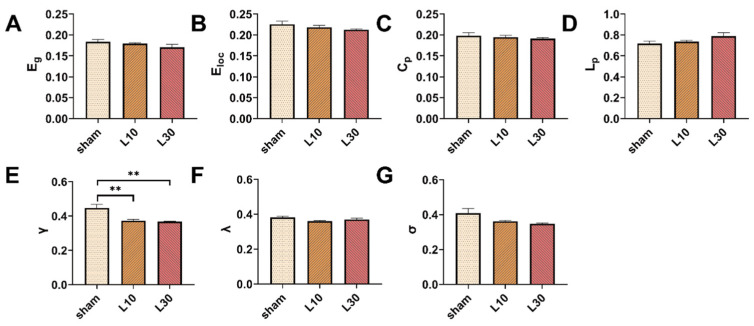
The effects of acute 1.5 GHz MW exposure on the topological organization of the rat brain network 1 d after the exposure. (**A**–**G**) show the comparisons of the whole-sparsity-range AUC of the brain network topological parameters (E_g_, E_loc_, C_p_, L_p_, γ, λ, and σ) among different groups (sham, L10, and L30 groups, *n* = 5) 1 d after MW radiation. Data are shown as mean ± SEM. ** *p* < 0.01, vs. sham. Abbreviations: MW, microwave; E_g_, global efficiency; E_loc_, local efficiency; C_p_, clustering coefficient; L_p_, characteristic path length; γ, normalized clustering coefficient; λ, normalized characteristic path length; σ, small-worldness; AUC, area under the curve.

**Figure 9:**
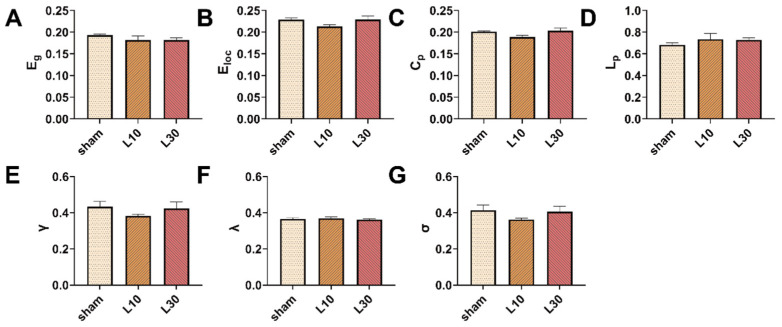
The effects of acute 1.5 GHz MW exposure on the topological organization of the rat brain network 7 d after exposure. (**A**–**G**) show the comparisons of the whole-sparsity-range AUC of the brain network topological parameters (E_g_, E_loc_, C_p_, L_p_, γ, λ, and σ) among different groups (sham, L10, and L30 groups, *n* = 5) 7 d after MW radiation. Data are shown as mean ± SEM. Abbreviations: MW, microwave; E_g_, global efficiency; E_loc_, local efficiency; C_p_, clustering coefficient; L_p_, characteristic path length; γ, normalized clustering coefficient; λ, normalized characteristic path length; σ, small-worldness; AUC, area under the curve.

To further quantitatively explore the effects of MW exposure on the topological organization of the rat brain network, the AUCs of the brain network parameters across the whole range of sparsity thresholds (0.15, 0.5) were compared among different groups. One day after 10 mW/cm^2^ and 30 mW/cm^2^ 1.5 GHz MW exposure, there was a significant difference in γ values among the sham, L10, and L30 groups [F (2, 12) = 10.342, *FDR* = 0.014]. As shown in Figure 8E, the result of the post hoc test indicates that the γ values of rats in both of the L10 group and L30 group were significantly decreased (L10 group: 0.372 ± 0.008, *p* = 0.003; L30 group: 0.368 ± 0.003, *p* = 0.002) compared with that in the sham group (0.446 ± 0.022). On the contrary, for other topological parameters, no significant differences were observed among the sham, L10, and L30 groups 1 d after the MW exposure. Seven days after 10 mW/cm^2^ and 30 mW/cm^2^ 1.5 GHz MW exposure, no significant differences in the topological parameters of the rat brain network were observed among the sham, L10, and L30 groups (Figure 9).

Taken together, these results suggested that acute exposure to both 10mW/cm^2^ and 30mW/cm^2^ 1.5 GHz MW could induce disrupted topological organization of the brain network in rats 1 d after the exposure. These adverse effects were recovered 7 d after the exposure.

### 3.5. Acute Exposure to 10 mW/cm^2^ or 30 mW/cm^2^ 9.375 GHz MW Induced No Adverse Effects on Topological Organization of the Brain Network in Rats

**Figure 10:**
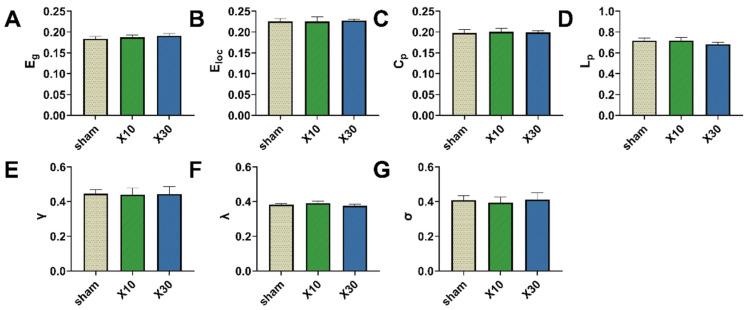
The effects of acute 9.375 GHz MW exposure on the topological organization of the rat brain network 1 d after exposure. (**A**–**G**) show the comparisons of the whole-sparsity-range AUC of the brain network topological parameters (E_g_, E_loc_, C_p_, L_p_, γ, λ, and σ) among different groups (sham, X10, and X30 groups, *n* = 5) 1 d after MW radiation. Data are shown as mean ± SEM. Abbreviations: MW, microwave; E_g_, global efficiency; E_loc_, local efficiency; C_p_, clustering coefficient; L_p_, characteristic path length; γ, normalized clustering coefficient; λ, normalized characteristic path length; σ, small-worldness; AUC, area under the curve.

**Figure 11:**
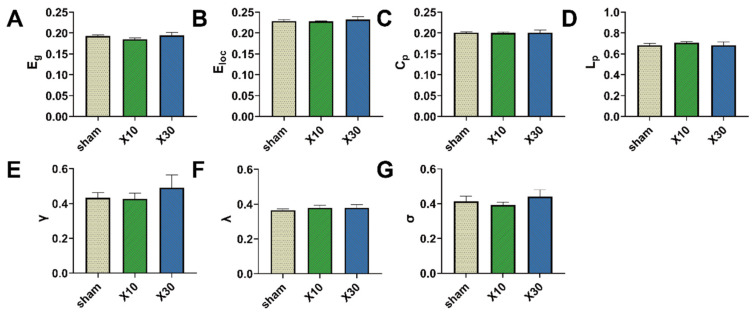
The effects of acute 9.375 GHz MW exposure on the topological organization of the rat brain network 7 d after exposure. (**A**–**G**) show the comparisons of the whole-sparsity-range AUC of the brain network topological parameters (E_g_, E_loc_, C_p_, L_p_, γ, λ, and σ) among different groups (sham, X10, and X30 groups, *n* = 5) 7 d after MW radiation. Data are shown as mean ± SEM. Abbreviations: MW, microwave; E_g_, global efficiency; E_loc_, local efficiency; C_p_, clustering coefficient; L_p_, characteristic path length; γ, normalized clustering coefficient; λ, normalized characteristic path length; σ, small-worldness; AUC, area under the curve.

One day after 10 mW/cm^2^ and 30 mW/cm^2^ 9.375 GHz MW exposure, across the whole range of sparsity thresholds (0.15, 0.5), no obvious changes in the topological parameters were observed in rats in the X10 and X30 groups compared with rats in the sham group (Figure 6 and Figure 7). Moreover, as shown in Figure 10 and Figure 11, 1 d or 7 d after the MW exposure, there were no significant differences in the AUCs of topological parameters among the sham, X10, and X30 groups. These results suggest that acute exposure to 10 mW/cm^2^ or 30 mW/cm^2^ 9.375 GHz MW induced no adverse effects on the topological organization of the brain network in rats.

## 4. Discussion

In this study, for the first time, we investigated the topological organization of the brain network in rats with spatial memory impairments induced by acute MW radiation. Our results demonstrated that the rats exposed to 30 mW/cm^2^ 1.5 GHz MW radiation exhibited brain network topological organization disruptions 1 d after exposure and spatial memory deficits 3 d after exposure. Moreover, after 10 mW/cm^2^ 1.5 GHz MW radiation, brain network topological organization disruptions were also observed in rats without any changes in spatial memory. On the other hand, no adverse effects on spatial memory ability or brain network topological organization were observed after 30 mW/cm^2^ or 10 mW/cm^2^ 9.375 GHz MW radiation, which might be attributed to the relatively lower energy deposit and lesser temporal variation in the energy deposit in the rat brains [54,55]. The findings of this work further our understanding of the mechanism underlying acute-MW-exposure-induced spatial memory impairments. Meanwhile, we also demonstrated the possibility of using topological parameters of the functional brain network as early and sensitive diagnostic indicators.

Previous studies suggested that MW with certain parameters can induce spatial memory deficits [56,57]. Wang et al. [41] found that compared to the sham group, the AEL of rats subjected to 1.5 GHz MW exposure significantly increased, which indicated a decline in spatial memory ability. Tan et al. [58] also reported that exposure to 1.5 GHz MW could impair the spatial memory of rats. In this study, consistent with the findings of previous studies, our results demonstrated that the AEL of rats in an MWM was significantly prolonged after 30 mW/cm^2^ 1.5 GHz MW exposure. These results suggested that acute exposure to 30 mW/cm^2^ 1.5 GHz MW can induce spatial memory impairments in rats.

Previous pioneering studies have already demonstrated the adverse effects of MW on regional brain activity and local functional connectivity using rs-fMRI [15,16]. However, the brain works as a tightly integrated and highly efficient network rather than isolated brain regions. The resting-state brain network is topologically organized according to a small-world architecture that not only permits the complex information segregation and integration during high cognitive processes, such as memory, but also determines the clinical consequences of alterations encountered in development, ageing, or neurological diseases [59,60]. Therefore, investigations of the topological organization properties of the integrative brain network can provide us with new insights into large-scale brain-communication changes induced by MW radiation [17]. In this study, for the first time, we found that both 10 mW/cm^2^ and 30 mW/cm^2^ 1.5 GHz MW could induce a significant decrease in γ in rats. These results suggest that acute 1.5 GHz MW exposure can induce disruptions in the functional brain network topological organization.

Changes in the overall topological organization of the resting-state brain network are directly linked to memory deficits [61]. γ indicates efficiency in local information processing [62,63]. Zhang et al. [38] found that the γ of the brain network was significantly decreased in renal-disease patients with memory impairments. Sinha et al. [64] also found that increased γ was correlated with improvements in the clinical symptoms and associated memory deficits of depression patients. In this study, significant spatial memory impairments, along with significantly decreased γ, were observed in the rats subjected to 30 mW/cm^2^ 1.5 GHz MW exposure. These results suggest that memory deficits induced by MW radiation might rely on a decline in local information processing and loss of small-worldness in the brain network.

Furthermore, our results also demonstrate the potential of using topological parameters of the brain network in the early detection of acute-MW-exposure-induced spatial memory impairments. It has been reported that the topological parameters of the brain network could be used as early indicators of cognitive dysfunctions. Drakesmith et al. [65] reported that the network topological parameters could act as a sensitive biomarker of subclinical schizophrenia and a potential predictor for forecasting the disease’s development, indicating cognitive dysfunctions in severe forms of the condition. Tuo et al. [66] observed that the topological organization properties of the brain network in patients with carotid plaques were significantly changed, while their cognitive functions remained normal in the early stage. They concluded that these changes in the brain network may emerge before subjective symptom onset. In this study, we found that the rats subjected to 30 mW/cm^2^ 1.5 GHz acute MW exposure exhibited a disruption in the brain network organization 1 d after exposure, occurring 2 days earlier than the appearance of the behavior symptom of spatial memory deficits. Moreover, the rats exposed to 10 mW/cm^2^ 1.5 GHz MW merely demonstrated disruptions in the brain network organization, without any changes in spatial-memory-related behavioral performance. These results suggest that the disruptions in the topological organization of the rat brain network emerged before behavioral symptom onset and could even be found in rats without a decline in the performance of spatial memory tasks. Therefore, the topological parameters of the rat brain network could be used as potential early and sensitive indicators of acute-MW-radiation-induced spatial memory impairments, a finding of great significance for corresponding early detection methods and interventions.

In addition, no significant changes in the spatial memory or topological organization of the brain network were observed in the rats subjected to 9.375 GHz MW exposure. This might be attributed to the specific spatial distributions of and temporal variations in the energy deposits in the rat brains depending on the frequencies of MW. SAR is defined as the rate at which energy is deposited in a biological subject [67]. The dose-dependent relationship between MW radiation and its effects on brain functions and related behaviors have long been acknowledged. Gokcek-Sarac C [68] reported the potential dose-dependent effects of 2.1 GHz MW on rat hippocampus-related behavioral performance as well as the levels of cholinergic biomarkers in the hippocampus. Wang [69] suggested that continuous MW exposure could cause dose-dependent long-term impairments in spatial memory and hippocampal structure injuries. In this study, our results demonstrated that the spatial distributions of the SAR values of rat brains depended on the frequencies of MW. For 1.5 GHz MW, the energy was mainly deposited in the brain. On the other hand, for 9.375 GHz MW, the energy was mainly deposited in the dorsal skin of the head. As a result, with the same incidence-averaged power density, the average SAR values of the rat brains at 1.5 GHz MW exposure (13.458 W/kg for 10 mW/cm^2^; 40.376 W/kg for 30 mW/cm^2^) were approximately four-fold those at 9.375 GHz exposure (3.375 W/kg for 10 mW/cm^2^; 10.125 W/kg for 30 mW/cm^2^). Moreover, considering the rotation of the rats during exposure, we also examined the time variations in the SAR values of the rat brains. The results demonstrate that 1.5 GHz MW resulted in much larger temporal variations in the SAR values (80.254% for 10 mW/cm^2^; 80.252% for 30 mW/cm^2^) than 9.375 GHz MW (20.267% for 10mW/cm^2^; 20.278% for 30 mW/cm^2^). Therefore, the fact that only 1.5 GHz MW induced significant disturbances in spatial memory abilities and disruptions of the brain network organization might be explained by the higher energy deposits in the rat brains and their larger temporal variations compared with 9.375 GHz MW.

There were several limitations in the present study. Firstly, in this study, the circular exposure area was only divided into eight fan-shaped zones. Within each zone, the SAR values of rat brains at different E-polarization angles were assumed to be the same as that obtained at the center E-polarization angle of the zone. If we divide the circular exposure area into more zones, the assumption will be more solid and the approximate average SAR value in the rat brain will be closer to the real value. Secondly, in the rs-fMRI experiments performed in this study, the rats were anesthetized using sodium pentobarbital to minimize their stress and movement during rs-fMRI scanning. However, it has been reported that anesthesia can affect the properties of the rat brain network [70]. Though the sham group was implemented in this work to exclude the effects of other conditions (such as anesthesia) besides MW exposure, the anesthesia method, which has less impact on the resting-state brain network [71], could be used in future studies to further minimize such influences.

## 5. Conclusions

In conclusion, rats with spatial memory deficits induced by MW radiation exhibited disruptions in the topological organization of the brain network. Moreover, these topological-organization disruptions emerged earlier than the behavior symptom onset and could even be found in rats without a behavioral performance decline, which suggests their potential value as an early and sensitive indicator of acute-MW-radiation-induced spatial memory impairments.

## Figures and Tables

**Table 1:** The SAR values of rat brains with different center E-polarization angles (0°, 45°, 90°, 135°, 180°, 225°, 270°, and 315°) and the approximate average brain SAR values $\bar{{SAR}_{brain}}\prime$ of different exposure groups.

	SAR_*brain*,0°_ (W/kg)	SAR_*brain*,45°_ (W/kg)	SAR_*brain*,90°_ (W/kg)	SAR_*brain*,135°_ (W/kg)	SAR_*brain*,180°_ (W/kg)	SAR_*brain*,225°_ (W/kg)	SAR_*brain*,270°_ (W/kg)	SAR_*brain*,315°_ (W/kg)	$\bar{{SAR}_{brain}}\prime$ (W/kg)
L10	18.859	13.549	8.058	13.368	18.859	13.549	8.058	13.368	13.458
L30	56.578	40.647	24.175	40.106	56.578	40.647	24.175	40.106	40.376
X10	3.717	3.351	3.033	3.399	3.717	3.351	3.033	3.399	3.375
X30	11.151	10.052	9.098	10.197	11.151	10.052	9.098	10.197	10.125

## Data Availability

The experimental data used to support the findings of this study are included in the article.
